# Enantioselective Biosynthesis of l-Phenyllactic Acid by Whole Cells of Recombinant *Escherichia coli*

**DOI:** 10.3390/molecules22111966

**Published:** 2017-11-15

**Authors:** Yibo Zhu, Ying Wang, Jiayuzi Xu, Jiahao Chen, Limei Wang, Bin Qi

**Affiliations:** 1 School of Biotechnology and Food Engineering, Changshu Institute of Technology, Changshu 215500, China; centuryrain@cslg.edu.cn (Y.Z.); 15895618691@163.com (Y.W.); XJM337463903@163.com (J.X.); 15606238552@163.com (J.C.); wlmqb@126.com (L.W.); 2 Key Laboratory of Food and Biotechnology of Suzhou, Changshu Institute of Technology, Changshu 215500, China; 3 College of Food Science and Engineering, Jilin Agricultural University, Changchun 130118, China

**Keywords:** l-Phenyllactic acid, l-Lactate dehydrogenase, glucose dehydrogenase, NADH regeneration, whole-cell transformation

## Abstract

Background: l-Phenyllactic acid (l-PLA)—a valuable building block in the pharmaceutical and chemical industry—has recently emerged as an important monomer in the composition of the novel degradable biocompatible material of polyphenyllactic acid. However, both normally chemically synthesized and naturally occurring phenyllactic acid are racemic, and the product yields of reported l-PLA synthesis processes remain unsatisfactory. Methods: We developed a novel recombinant *Escherichia coli* strain, co-expressing l-lactate dehydrogenase (l-LDH) from *Lactobacillus plantarum* subsp. *plantarum* and glucose dehydrogenase (GDH) from *Bacillus megaterium*, to construct a recombinant oxidation/reduction cycle for whole-cell biotransformation of phenylpyruvic acid (PPA) into chiral l-PLA in an enantioselective and continuous manner. Results: During fed-batch bioconversion with intermittent PPA feeding, l-PLA yield reached 103.8 mM, with an excellent enantiomeric excess of 99.7%. The productivity of l-PLA was as high as 5.2 mM·h^−1^ per OD_600_ (optical density at 600 nm) of whole cells. These results demonstrate the efficient production of l-PLA by the one-pot biotransformation system. Therefore, this stereoselective biocatalytic process might be a promising alternative for l-PLA production.

## 1. Introduction

The development of novel natural antimicrobial compounds to preserve food and feed has gained importance in recent years due to the increasing demand for chemical preservative-free products by consumers and the increased interest in food biopreservation [1,2]. 3-Phenyllactic acid (2-hydroxy-3-phenylpropanoic acid; PLA)—a special chemical widely used for the synthesis of chiral pharmaceutical and biorefinery chemicals, including bio-based polymers [3]—has been recently identified as a novel natural antiseptic agent with broad-spectrum and effective inhibitory activity against a variety of food-borne microorganisms, including a wide range of bacteria and fungi [4,5,6,7,8]. In addition, PLA is the monomer for synthesis of the novel biocompatible poly(phenyllactide). Due to containing an aromatic ring, PLA and its dimer are particularly interesting monomers with different properties than those of polylactide [9,10]. As a result of a chiral carbon atom in its molecule, PLA occurs as two enantiomers, l-3-phenyllactic acid (l-PLA) and d-3-phenyllactic acid (d-PLA), both of which are valuable chiral building blocks but have different biological functions, leading to their various applications in the pharmaceutical and chemical industries [11]. d-PLA is applied in the synthesis of hypoglycemic drugs and high-efficiency and low-toxicity anthelmintics, while l-PLA is an important precursor for the synthesis of the non-protein amino acid statine, anti-HIV drugs, and biodegradable poly(PLA), clearly indicating that the optical purity of stereoisomeric monomers plays a very important role in their applications [3,12,13,14]. Especially for food and pharmaceutical purposes, chiral monomers with high optical purity and low impurity levels are a prerequisite to avoid toxicosis and isomeric ballast [15]. Therefore, the preparation of optically pure chiral isomers has been one of the central subjects in pharmaceutical and food industries and chiral chemical production.

Various approaches for preparing PLA have been developed in recent years, including traditional chemical methods, enzymatic routes, and biological methods [16,17,18,19,20]. Through traditional chemical synthesis methods, chiral isomers have been produced with high yields and high conversion rates, but at the expense of the environment and extreme reaction conditions. Even chemical processes for chiral isomer production results in the racemic mixture of both stereospecific forms. Contrary to chemical synthesis, biocatalytic asymmetric synthesis has recently aroused great interest among industry and academia owing to its high conversion yield, high efficiency, simple operation, environment-friendly nature, and, in particular, its excellent stereoselectivity [21,22]. In addition, complex processes for enzyme separation and purification can be omitted by utilizing microbial whole cells as a biocatalyst, as well as the supplementary addition of cofactors and cofactor regeneration enzymes. Therefore, microbial whole-cell biocatalysis might be recognized as the most promising approach applicable for the asymmetric synthesis of chiral isomers. Thus far, the use of natural microorganisms to produce PLA has been well-studied in lactic acid bacteria, such as *Lactobacillus* [6,23,24,25], *Enterococcus* [7], and *Leuconostoc* [19,26]. However, few studies have reported l-PLA production by means of asymmetric synthesis by microorganisms, unlike d-PLA [27,28,29].

**Figure 1:**
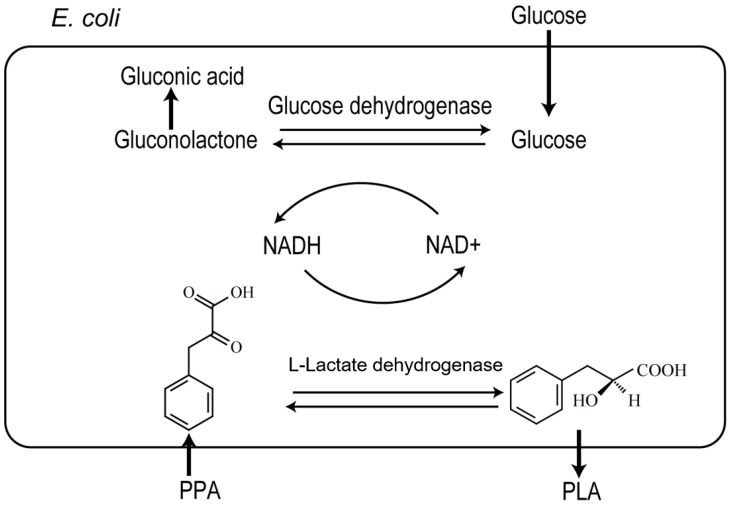
Scheme for the production of l-PLA from PPA by the NADH-dependent l-LDH and GDH coupling system. NADH represents the reduced form of nicotinamide adenine dinucleotide.

Here, we report the development of a recombinant *E. coli* strain co-expressing *l*-lactate dehydrogenase (*l-ldh*) from *Lactobacillus plantarum* subsp. *plantarum* and glucose dehydrogenase (*gdh*) from *Bacillus megaterium* in an inducible overexpression vector to construct a recombinant oxidation/reduction cycle for whole-cell biotransformation of phenylpyruvic acid (PPA) to chiral l-PLA (Figure 1). Enzymatic activity and the enantioselective reduction of PPA to l-PLA were evaluated. An efficient and desirable bioconversion process for l*-*PLA production by the regeneration of the in situ cofactor nicotinamide adenine dinucleotide (NADH) was developed.

## 2. Results

### 2.1. Cloning and Co-Expression of l-ldh and gdh in E. coli

The PCR products matched the length of *l-ldh* (963 bp) and *gdh* (786 bp). Nucleotide sequencing indicated that the cloned *l-ldh*, encoding 320 amino acids, has a 100% similarity with the nucleotide sequence of *L. plantarum* ATCC 14917 *l-ldh* (GenBank No. ACGZ02000027) and that the cloned *gdh*, encoding 261 amino acids, has a 99% similarity with the nucleotide sequence of *B. megaterium* WSH-002 *gdh* (GenBank No. NC 017138).

**Figure 2:**
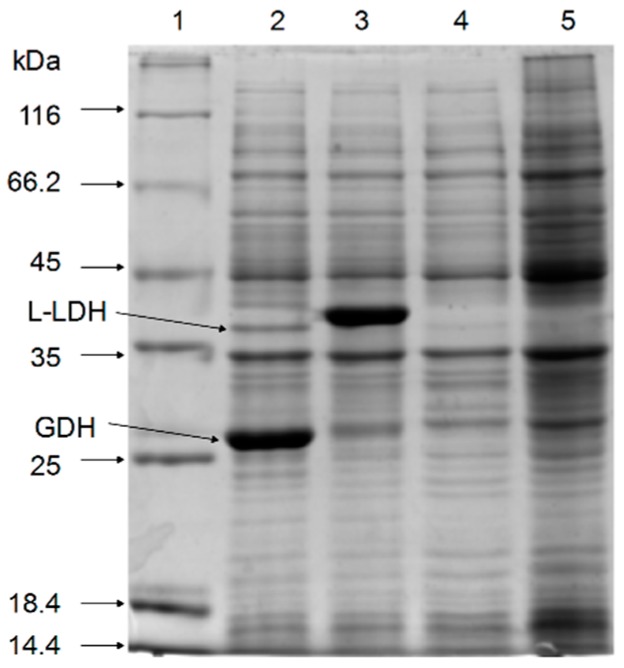
Validation of the expression of l-LDH and GDH in induced recombinant *E. coli*. Lane 1: protein marker; lane 2: *E. coli* pETDuet-*ldhL-gdh*; lane 3: *E. coli* pETDuet-*ldhL*; lane 4: *E. coli* pETDuet-1; lane 5: *E. coli* BL21 (DE3).

SDS-PAGE of the cell-free extracts showed that the recombinant l-LDH (40 kDa) and GDH (27 kDa) proteins were co-expressed successfully in recombinant *E. coli* strains (Figure 2).

### 2.2. Enzyme Assays of l-LDH and GDH

One of the most successful NADH regeneration systems is glucose dehydrogenase (GDH; E.C.1.1.1.47) owing to its high activity and its substrate glucose, which is a cheap source of reducing equivalent. For the replenishment of the intracellular NADH pool, which decreased during whole-cell bioconversions with resting cells, we attempted the overexpression of *gdh* under the control of the lac promoter in the pET system. GDH activity was detected in strain *E. coli* pETDuet-*ldhL-gdh* (5.37 ± 0.74 U·mg^−1^) but not in *E. coli* pETDuet-*ldhL* and *E. coli* pETDuet-1 (Table 1), which indicated that GDH was successfully expressed in *E. coli* pETDuet-*ldhL-gdh*. Unlike *E. coli* pETDuet-1, both *E. coli* pETDuet-*ldhL* and *E. coli* pETDuet-*ldhL-gdh* showed LDH activity (Table 1), indicating that l-LDH was successfully expressed in both strains and had catalytic function.

### 2.3. Optimization of Bioconversion Conditions

**Figure 3:**
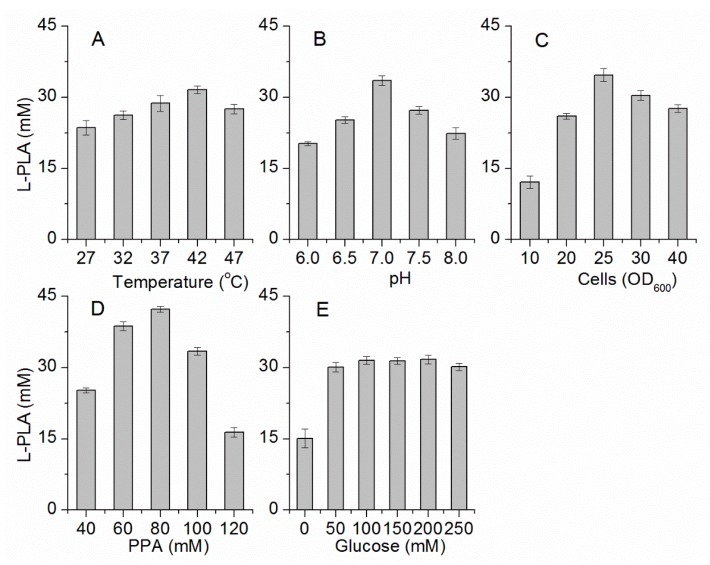
Optimization of bioconversion conditions. (**A**) Temperature; (**B**) pH; (**C**) Concentration of whole cells; (**D**) Concentration of PPA; (**E**) Concentration of glucose.

To increase the efficiency of l-PLA production by *E. coli* pETDuet-*ldhL-gdh*, the biocatalytic factors were optimized for the concentrations of PPA, glucose, and whole cells; temperature; and pH. First, the highest l-PLA production was detected at 42 °C (Figure 3A). Within the range of the thermal tolerance of *E. coli*, the high temperature increased the solubility and dissolution rate of PPA, contributing to effective conversion of PPA into PLA at a high concentration and with high productivity. Then, because pH would considerably affect the enzyme activity and stability, the effect of pH on this l-LDH and GDH coupling system was also investigated. l-PLA production was the highest at pH 7.0 (Figure 3B). Under acidic or alkaline conditions, the bioconversion of PPA to l-PLA decreased. Next, taking economic efficiency into consideration, the optimal concentration of cells was determined to be OD_600_ = 25 (Figure 3C). Furthermore, the optimal PPA concentration was determined to be 80 mM (Figure 3D), while the concentration of the co-substrate (glucose) between 100 mM to 200 mM elicited no apparent influence on l-PLA production (Figure 3E). In addition, the l-PLA production had a significant improvement in the presence of glucose (Figure 3E). Thus, the concentration of glucose was determined to be 100 mM for the following experiments.

### 2.4. Enhancing l-PLA Production by a Cofactor Regeneration System

**Figure 4:**
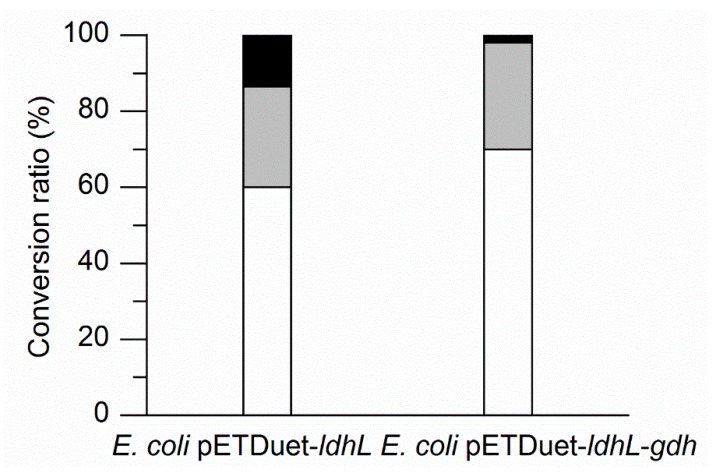
Comparison of batch biotransformation of l-PLA by *E. coli* pETDuet-*ldhL* and *E. coli* pETDuet-*ldhL-gdh* whole cells. White indicates l-PLA; black indicates residual PPA; and grey indicates PPA involved in other metabolic routes but PLA.

To identify the enhancement of l-PLA production by a cofactor regeneration system, *E. coli* pETDuet-*ldhL* or *E. coli* pETDuet-*ldhL-gdh* whole cells were used as the biocatalyst, respectively, in the batch bioconversion under optimal conditions. The processes were performed in 100 mL flasks with 10 mL of a 100 mM sodium phosphate buffer (pH 7.0) containing 50 mM PPA, 100 mM glucose, and *E. coli* pETDuet-*ldhL* or *E. coli* pETDuet-*ldhL-gdh* whole cells (OD_600_ = 25) at 42 °C and 200 rpm. After 40 min bioconversion, 68.80% of PPA was converted into l-PLA by *E. coli* pETDuet-*ldhL-gdh*, while only 58.92% of PPA was converted into l-PLA by *E. coli* pETDuet-*ldhL* (Figure 4). Moreover, 97.83% of PPA was consumed by *E. coli* pETDuet-*ldhL-gdh* and only 86.50% by *E. coli* pETDuet-*ldhL*. These differences suggested that the strain with the recombinant NADH regeneration system showed a higher PLA production rate and higher PPA consumption yield than that without a sufficient cofactor regeneration system.

In addition, higher l-LDH activity (Table 1) but lower l-PLA production ability (Figure 4) by *E. coli* pETDuet-*ldhL* in comparison with *E. coli* pETDuet-*ldhL-gdh* indicated the crucial importance of an in situ NADH regeneration system. On the other hand, up to 30% of PPA was not converted into PLA but other metabolites (Figure 4). Redesigning of the wild-type l-LDH gene and eliminating pathways that compete for NADH utilization by the knockout of d-LDH gene and other genes could improve l-PLA production.

### 2.5. Fed-Batch Bioconversion of l-PLA

**Figure 5:**
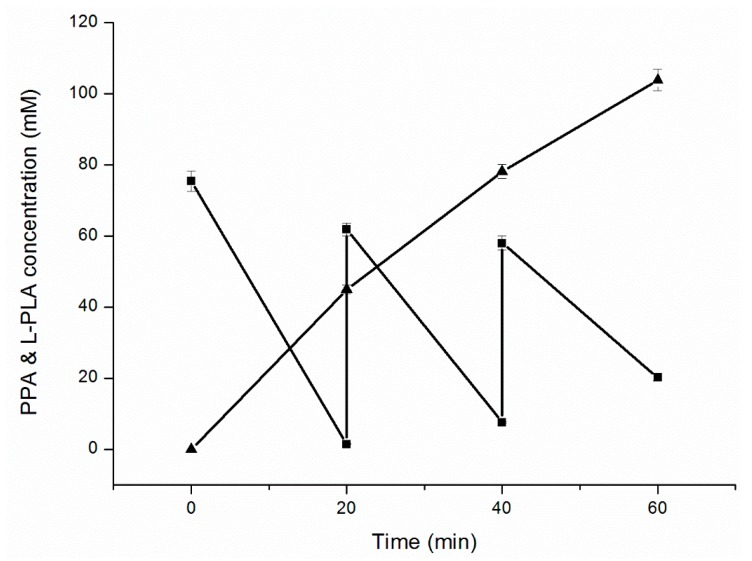
Time course of l-PLA production in fed-batch bioconversion with intermittent substrate feeding. (■) Concentration of PPA; (▲) Concentration of l-PLA.

Fed-batch bioconversion was performed with intermittent PPA feeding to increase PLA yield and avoid substrate inhibition. After 60 min of reaction (Figure 5), 103.8 mM of l-PLA with 99.7% *ee* was achieved from 186.2 mM of PPA, with a high productivity of 103.8 mM·h^−1^ and final conversion ratio of 55.8% (Figure 5).

**Figure 6:**
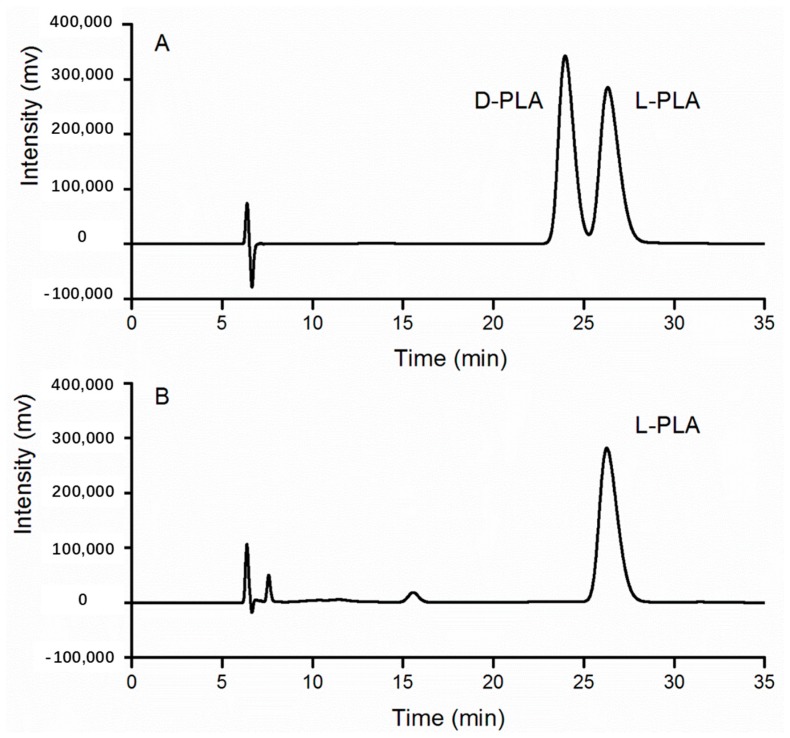
HPLC chiral analysis of PLA produced by whole cells of *E. coli* pETDuet-ldhL-gdh. (**A**) Standards of l-PLA and d-PLA; (**B**) Sample of conversion.

Furthermore, the bioconversion sample catalyzed by recombinant *E. coli* pETDuet-*ldhL-gdh* whole cells was proven to be l-PLA on the basis of the same retention time as that of the standard sample containing d-PLA and l-PLA at 26 min (Figure 6).

## 3. Discussion

l-PLA with an *ee* of 75% was obtained through whole-cell bioconversion of racemic 3-phenyllactonitrile using a *Pseudomonas* sp. BC-18 strain [13]. When fructose was used as the carbon source, *Rubrivivax benzoatilyticus* JA2 yielded a maximum of 0.92 mM l-PLA from l-phenylalanine (1 mM) [30]. Thus, further application of l-PLA has been limited by the unsatisfactory stereoselectivity and low production ability of known l-PLA-producing strains. It has, therefore, been deemed imperative for large-scale production of l-PLA to use high-producing strains [5].

As a paradigm organism for metabolic engineering, *E. coli* does not possess a sufficient cofactor regeneration system. Most of the widely applied oxidoreductases require cofactors for their catalytic activity. The representative cofactor NADH plays a central role in cellular metabolism in over 300 reduction–oxidation reactions [31], including the asymmetric reduction of PPA into l-PLA by NADH-dependent l-LDH in this study. Therefore, in cofactor-dependent biotransformation systems, if the enzyme is overexpressed and the enzyme level is not the bottleneck of the conversion, cofactor availability and the ratio of the reduced to oxidized form of the cofactor may become limiting factors; in this situation, cofactor manipulation is imperative for high productivity [32]. The regeneration of NADH based on formate/formate dehydrogenase has been developed to the most advanced level [33]. However, the formate dehydrogenase system has drawbacks, especially for industrial applications, such as only moderate stability and relatively low specific activity. Another general coenzyme NADH regenerator, GDH, is a low-cost, highly active (>250 U·mg^−1^), and stable [34] option. GDH catalyzes the oxidation of β-d-glucose to β-d-glucono-1,5-lactone, coupled with the simultaneous reduction of the cofactor NAD(P)^+^ to NAD(P)H [35]. Because of the rapid hydrolysis of unstable gluconolactone to gluconic acid, the GDH catalytic reaction is nearly irreversible, which provides a strong driving force for NADH regeneration [36]. Recently, 79.6 mM l-PLA (*ee* > 99%) was produced by using whole cells (OD_600_ = 50) of recombinant *E. coli* co-expressing NAD-dependent l-lactate dehydrogenase (l-nLDH) from *Bacillus coagulans* NL01 and formate dehydrogenase from *Candida boidinii* after 40 min of batch bioconversion [37]. In our study, during the first 20 min of fed-batch bioconversion (Figure 4), 45.0 mM l-PLA was produced with a bioconversion ratio of 59.9% by using whole cells (OD_600_ = 25) of recombinant *E. coli* pETDuet-*ldhL-gdh* co-expressing l-lactate dehydrogenase from *L. plantarum* and GDH from *B. megaterium*. A high bioconversion temperature of 42°C contributed to the rapid dissolution of PPA and high metabolic activity [20]. Per OD_600_ of *E. coli* pETDuet-*ldhL-gdh*, the l-PLA productivity was 5.2 mM·h^−1^, which was 1.26 times higher than that of *E. coli* reported by Zheng [37]. Nevertheless, the general conversion ratio of 55.8% (103.8 mM l-PLA from 186.2 mM PPA) of this study was much less than the ratio of 96% of the report. The blast result of l-nLDH (Access number: KF386111) and l-LDH of this study (Access number: EFK28653) indicated that the two enzymes contain the identical substrate binding site residues and active site, with 153 of 309 identities and 217 of 309 positives. Whether the different origins of the two enzymes led to the differences in the conversion ratio needs to be further studied.

In conclusion, a one-step bioconversion of PPA to l-PLA by whole cells of recombinant *E. coli* harboring heterogeneous *l-ldh* and *gdh* was successfully developed. l-PLA was efficiently produced by the one-pot biotransformation system. The yield of l-PLA reached 103.8 mM with an excellent *ee* of 99.7% and high productivity of 103.8 mM·h^−1^. The novel biocatalysis process has great potential as an alternative for the production of highly optically pure l-PLA at a high productivity and high stereoselectivity.

## 4. Materials and Methods

### 4.1. Enzymes and Chemicals

Restriction enzymes, Ex Taq DNA polymerase, T4 DNA ligase, and the pMD19-T simple, (TaKaRa, Dalian, China) cloning vector were purchased from TaKaRa Biotechnology Co. Ltd. (Dalian, China). The expression vector pET-Duet-1 was obtained from Novagen (Madison, WI, USA). PCR-related products, PCR primers, isopropyl-β-d-thiogalactopyranoside (IPTG), NADH, NAD^+^, and ampicillin were purchased from Sangon Biotech Co. Ltd. (Shanghai, China). B-Per^®^ reagent was obtained from Pierce (Rockford, IL, USA). PPA in the chemical form of sodium phenylpyruvate, l-PLA, and d-PLA were purchased from Sinopharm Chemical Reagent Co. Ltd. (Shanghai, China). All other chemicals were of analytical grade and were sourced commercially.

### 4.2. Bacterial Strains, Plasmids, and Growth Conditions

The bacterial strains, plasmids, and primers used in this study are listed in Table 2. *E. coli* DH5α was used as the cloning host. *E. coli* BL21 (DE3) was used for recombinant protein expression and whole-cell biotransformation. The pMD19-T (Simple) cloning vector was used for gene cloning. The pETDuet-1 plasmid used for gene expression harbors two multiple cloning sites, both of which were preceded by the T7 promoter, *lac* operator, and ribosome-binding site. *L. plantarum* was routinely cultured at 37 °C without shaking in de Man Rogosa Sharpe (MRS) broth. *E. coli* strains and *B. megaterium* were cultivated at 37 °C in Luria–Bertani (LB) medium or on agar plates, and ampicillin was added at a final concentration of 100 μg·mL^−1^, if necessary.

### 4.3. Cloning and Expression of l-ldh and gdh in E. coli BL21(DE3)

All DNA manipulations and bacterial transformations were carried out according to standard protocols and instructions. Genomic DNAs of *L. plantarum* CGMCC 1.2437 and *B. megaterium* CCTCC M2013244 were extracted as the templates for *l-ldh* and *gdh* PCR amplification, respectively. The *l-ldh* gene was amplified by PCR using forward primer P1 with a *BamH*I restriction site insertion and reverse primer P2 with a *Pst*I restriction site insertion. The *gdh* gene was amplified by PCR using forward primer P3 with a *Nde*I restriction site insertion and reverse primer P4 with an *Xho*I restriction site insertion.

Purified PCR products were ligated into the pMD19-T (Simple) cloning vector, and the resulting plasmids were designated as pMD-*ldhL* and pMD-*gdh*, respectively. Both the recombinant cloning vectors were then sequenced (Sangon Biotech Co. Ltd., Shanghai, China) to verify that no mutations were introduced by PCR.

Next, to construct the recombinant co-expression plasmid pETDuet-*ldhL-gdh*, the recombinant plasmid pMD-*gdh* was digested with restriction enzymes *Nde*I and *Xho*I. Next, the gel-purified *gdh* fragment was ligated into the multiple cloning sites-2 of the pETDuet-1 vector that had been digested with the corresponding restriction enzymes. The resulting plasmid was designated as pETDuet-*gdh*. The recombinant plasmids pMD-*ldhL* and pETDuet-*gdh*, as well as the pETDuet-1 vector, were digested with restriction enzymes *BamH*I and *Pst*I. The gel-purified *l-ldh* fragments were then ligated into the multiple cloning sites-2 of pETDuet-*gdh* and pETDuet-1 vector, respectively. Thus, pETDuet-*ldhL* and pETDuet-*ldhL-gdh* were both constructed. Then, the recombinant expression plasmids pETDuet-*ldhL* and pETDuet-*ldhL-gdh* were transformed into competent *E. coli* BL21 (DE3), and positive clones were screened and confirmed by colony PCR and restriction enzyme digestion.

For protein expression, *E. coli* strains were incubated in LB medium containing 100 μg·mL^−1^ ampicillin, if necessary, with shaking (200 rpm) at 37 °C. When the culture reached an optical density of 1.2 at 600 nm, 1 mM IPTG was added to induce the overexpression of l-LDH and GDH proteins. After induction at 25 °C for 4 h, cells were harvested by centrifugation at 6000× *g* for 5 min at 4 °C, washed twice, and resuspended with 100 mM sodium phosphate buffer. The resultant suspensions were disrupted by B-Per^®^ cell lysis reagent at 30 °C for 10 min. Cell debris was removed by centrifugation at 12,000× *g* for 10 min at 4 °C. The obtained supernatants were used as cell-free extracts for crude enzyme activity assays and sodium dodecyl sulfate-polyacrylamide gel electrophoresis (SDS-PAGE).

### 4.4. Enzyme Assays

Enzyme activities were assayed by monitoring the changes in absorbance at 340 nm corresponding to NADH oxidation or NAD^+^ reduction at 30 °C using fresh cell-free extracts. l-LDH activities on PPA and pyruvate were assayed. The reaction mixture contained 100 mM sodium phosphate buffer (pH 7.0), 0.2 mM NADH, and 10 mM pyruvate or PPA [20]. GDH activity was measured by the reaction mixture containing 100 mM sodium phosphate buffer (pH 8.0), 2 mM NAD^+^, and 100 mM glucose. One unit of enzyme activity was defined as the amount of enzyme that catalyzed the oxidation or reduction of 1 μmol NADH per minute. The protein concentration of cell-free extracts was determined using Lowry’s method, with bovine serum albumin as a standard. Specific activity was expressed as units per milligram of protein.

### 4.5. Biocatalyst Preparation and Optimization of Whole-Cell Biotransformation Conditions

Recombinant strains were cultured in LB medium supplemented with 100 μg·mL^−1^ ampicillin at 37 °C and 200 rpm shaking. When the culture reached an OD_600_ of 1.2, 0.2 mM IPTG was added to induce gene expression at 25 °C. After a 6 h induction, cells were harvested by centrifugation from 100 mL of culture at 6000× *g* for 5 min at 4 °C and washed twice with 100 mM sodium phosphate buffer (pH 7.0). The pellets were used as whole-cell biocatalysts.

Batch biotransformation by whole cells was performed at 200 rpm for 20 min in 100 mL flasks with 10 mL of the reaction mixtures containing sodium phosphate buffer, PPA, glucose, and whole cells of recombinant *E. coli* pETDuet-*ldhL-gdh*. To improve the transformation efficiency of the l-LDH and GDH coupling system, the relevant conditions for biocatalysis were optimized. Different parameters were varied as follows: cell concentrations were set with an optical density of 10–40 at 600 nm; pH values of 100 mM sodium phosphate buffer were 6.0–8.0; PPA concentrations were 64–160 mM; glucose concentrations were 0–250 mM; and transformation temperatures were 27–47 °C. After 20 min of conversion, the samples were prepared by 5 min centrifugation at 10,000× *g* and 4 °C. The concentrations of PPA and PLA in the resulting supernatants were analyzed by HPLC. All experiments were performed in triplicate.

### 4.6. Fed-Batch Bioconversion of l-PLA with Substrate Feeding by the Cofactor Regeneration System

The fed-batch bioconversion process was performed in 250 mL flasks containing 20 mL of the reaction mixtures. The initial reaction mixtures containing 100 mM sodium phosphate buffer (pH 7.0), glucose (100 mM), PPA (75.4 mM), and cells (OD_600_ = 25) were incubated at 42 °C and 200 rpm for 60 min. PPA (2.5 mL 500 mM) and glucose powder (0.3 g) were both supplemented at 20 and 40 min by a pulse-feeding strategy.

### 4.7. Analytical Procedures

PPA and PLA were quantified by HPLC as reported earlier [27]. The stereoselective assay of PLA was performed by an HPLC system equipped with a CHIRALCEL OJ-RH column (150 × 4.6 mm) and a detector (SPD-20AV; Shimadzu, Kyoto, Japan) at 210 nm. The mobile phase was a water/methanol/acetonitrile solvent mixture (900:50:50, *v*/*v/v*) containing 0.15% trifluoroacetic acid at a flow rate of 0.3 mL·min^−1^ at 35 °C. The enantiomeric excess (*ee*) value of l-PLA is defined as (C_L-PLA_ − C_D-PLA_)/(C_L-PLA_ + C_D-PLA_) × 100%, where C_L-PLA_ and C_D-PLA_ represent the concentrations of l-PLA and d-PLA, respectively.

## Figures and Tables

**Table 1:** Specific activity of l-LDH and GDH of recombinant strains.

Strain	l-LDH Specific Activity (U mg^−1^)	GDH Specific Activity (U mg^−1^)
*E. coli* pETDuet-1	ND	ND
*E. coli* pETDuet-*ldhL*	28.11 ± 1.17	ND
*E. coli* pETDuet-*ldhL-gdh*	9.48 ± 0.91	5.37 ± 0.74

ND: not detected or specific activity <0.1 U mg^−1^.

**Table 2:** Strains, plasmids and primers used in this study.

Strain, Plasmid or Primer	Relevant Characteristics	Source
*Lactobacillus plantarum*	Wild type, source of *l-ldh*	CGMCC 1.2437
*Bacillus megaterium*	Wild type, source of *gdh*	CCTCC M2013244
*E. coli* DH5α	*φ*80 *lacZ*ΔM15Δ(*lac*ZYA-*arg*F) U169 *recA*1 *endA*1 *hsdR*17 *supE*44λ-thi-1	Novagen
*E. coli* BL21(DE3)	*F*^−^ *ompT hsdS_B_*(r_B_^−^m_B_^−^)*gal lon*λ*dcm* (DE3)	Novagen
*E. coli* pETDuet-1	*E. coli* BL21(DE3) harboring pETDuet-1	This study
*E. coli* pETDuet-*ldhL-gdh*	*E. coli* BL21(DE3) harboring pETDuet-*ldhL-gdh*	This study
*E. coli* pETDuet-*ldhL*	*E. coli* BL21(DE3) harboring pETDuet-*ldhL*	This study
pMD19-T (Simple)	Cloning vector, Amp ^r^	TaKaRa
pMD-*ldhL*	*l-ldh* in pMD19-T	This study
pMD-*gdh*	*gdh* in pMD19-T	This study
pETDuet-1	Expression vector, Amp ^r^	Novagen
pETDuet-*ldhL*	*l-ldh* in pETDuet-1	This study
pETDuet-*ldhL-gdh*	*l-ldh* and *gdh* in pETDuet-1	This study
P1 (*Bam*H I)	5’-AAGGGATCCATTGTCAAGCATGCCAAATC-3’	This study
P2 (*Pst* I)	5’-CGCCTGCAGGGCCATTATTTATTTTCTAATTCAG-3’	This study
P3 (*Nde* I)	5’-GCGCCCATATGATGTATAAAGATTTAGAAGG-3’	This study
P4 (*Xho* I)	5’-CTAGCTCGAGCATTATCCGCGTCCTGCTT-3’	This study

The underlined character indicates the restriction sites. ^r^ represents the resistance.
